# Wedging open a catalytic site

**DOI:** 10.7554/eLife.52418

**Published:** 2019-11-08

**Authors:** Annie Beuve

**Affiliations:** Department of Pharmacology, Physiology and Neuroscience, New Jersey Medical SchoolRutgers, the State University of New JerseyNewarkUnited States

**Keywords:** CryoEM, nitric oxide, cyclic GMP, manduca sexta, allostery, None

## Abstract

The activation mechanism of the nitric oxide receptor has been revealed by cryo-electron microscopy.

**Related research article** Horst BG, Yokom AL, Rosenberg DJ, Morris KL, Hammel M, Hurley JH, Marletta MA. 2019. Allosteric activation of the nitric oxide receptor soluble guanylate cyclase mapped by cryo-electron microscopy. *eLife* **8**:e50634. doi: 10.7554/eLife.50634

Of the many ways to regulate blood pressure, the simplest is to dilate or narrow the blood vessels. In the body, the gas nitric oxide (NO) binds to the enzyme soluble guanylyl cyclase (sGC) to relax blood vessels and decrease blood pressure. When the interaction between NO and sGC is disrupted, people develop hypertension and pulmonary arterial hypertension, and have a higher risk of heart failure. This makes sGC a major target for the treatment of cardiovascular diseases.

Drugs that activate the sGC enzyme have been used for many years, often without understanding their mechanism of action. One striking example is nitroglycerin, which has been used to treat angina pectoris since the end of the 19th century. It took over 100 years to discover that nitroglycerin and other nitrates work by generating NO or its derivatives, which then stimulate sGC to produce cyclic guanosine monophosphate (cGMP). The cGMP produced in this way relaxes the coronary arteries and increases blood flow to the heart. Unfortunately, nitroglycerin and most NO donors induce resistance, meaning that the patient becomes less and less responsive to ever-increasing doses of the drug.

In 1994, a small molecule called YC-1, which is not an NO derivative, was found to dramatically enhance the ability of the sGC enzyme to produce cGMP at low NO concentrations (Ko et al., 1994; Friebe et al., 1998). In healthy individuals, NO is produced by endothelial cells, which line the inside of blood vessels. When these cells stop performing their normal roles, the resulting low levels of NO lead to vascular diseases.

Nowadays, pulmonary arterial hypertension is treated with molecules similar to YC-1 (such as Adempas), which can stimulate the sGC enzyme without inducing resistance. These drugs are also in clinical trials for the treatment of chronic heart failure and have the potential to be used for treating chronic kidney diseases, hypertension and fibrotic diseases (Buys et al., 2018; Sandner et al., 2018). Two questions have been the subject of intense scrutiny and conflicting data: where does YC-1 bind to sGC, and how does it increase the impact of NO. Now, in eLife, Michael Marletta, Jim Hurley and colleagues at Berkeley, including Ben Horst and Adam Yokom as joint first authors, report the results of cryo-electron microscopy (cryo-EM) and small angle X-ray scattering experiments that shed light on these questions (Horst et al., 2019).

Structurally and kinetically, sGC is a complex enzyme. It is a heterodimer formed by two subunits, α and β, which each contain four domains: an N-terminal heme nitric oxide/oxygen binding (HNOX) domain; a Per/Arnt/Sim (PAS)-fold domain; a coiled-coil (CC) domain; and a catalytic domain at the C-terminal (Figure 1A). NO binds to the heme in the β subunit. Over the years, X-ray structures of each domain have been obtained, but attempts to determine the full-length 3D structure of the sGC enzyme have failed repeatedly. Without such structures it is not clear how these domains rearrange upon NO binding to create the most favorable conformation for the production of cGMP (Montfort et al., 2017; Horst and Marletta, 2018). Moreover, it was impossible to determine how molecules similar to YC-1 achieve their therapeutic effect.

**Figure 1.**
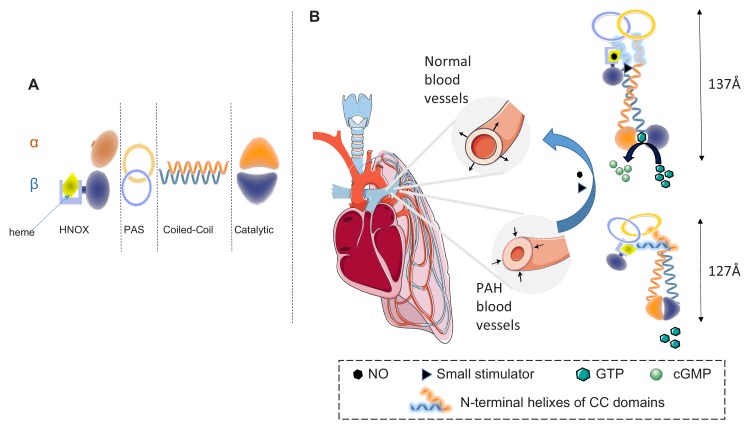
Soluble guanylyl cyclase (sGC) and the treatment of pulmonary arterial hypertension (PAH). (**A**) The enzyme sGC contains two subunits, α and β, and each subunit is composed of four domains: the N-terminal heme nitric oxide/oxygen binding (HNOX) domain, the Per-Arnt-Sim (PAS) domain, the coiled-coil (CC) domain, and the catalytic domain. (**B**) In pulmonary arterial hypertension blood vessels contract excessively, thus increasing arterial pressure (left); this process can be reversed (blue arrow) by nitric oxide (NO, hexagons) and drugs called small sGC stimulators (triangles). These molecules bind to sGC and change its conformation so it becomes active and can catalyze the conversion of guanosine triphosphate (GTP, green hexagons) to cyclic guanosine monophosphate (cGMP, light green circles). This last molecule acts a secondary messenger and induces the dilation of blood vessels. Horst et al. have shed light on the mechanism of activation for the sGC enzyme. In the inactive state (bottom), the catalytic site is inaccessible, so the enzyme cannot catalyze the reaction that converts GTP to cGMP; there is also a sharp bend in each coiled-coil domain. When NO binds to the heme in the HNOX domain of the β subunit, the HNOX and PAS domains rotate by 71° leading to the straightening of the bend in the two coiled-coil domains, which opens the catalytic site, thus allowing the enzyme to catalyze the conversion of GTP to cGMP (top). Small stimulators such as YC-1 bind into the space created between the β HNOX and CC domains, acting as a wedge that keeps the enzyme active for longer.

Horst et al. employed cryo-EM to obtain the full-length 3D structures of sGC in a non-activated state and in an activated state after the addition of NO and YC-1. Comparing these two structures reveals the structural changes caused by NO binding and suggest a likely mechanism for the therapeutic action of YC-1-like drugs. When NO binds to the sGC enzyme, a major rearrangement of the HNOX and PAS domains in the β subunit takes place, inducing the CC domains, which include a bend, to become straighter. This twist, in turn, creates an interface between the HNOX and CC domains in the β subunit. These conformational changes lead to opening of the catalytic pocket, making it possible for the sGC enzyme to bind guanosine triphosphate (GTP) and catalyze the production of cGMP (Figure 1B, right panel). Interestingly, YC-1 was found in a site created by the NO-induced rearrangement of the β HNOX and CC domains, where it could act as a wedge to maintain an unbent active conformation.

The structures reported by Horst et al. are similar to recent cryo-EM structures of sGC with and without excess NO (Kang et al., 2019). This suggests that activation at low NO concentrations enhanced by YC-1 is structurally comparable to that at high NO concentrations in the absence of YC-1. These models of sGC activation end years of frustration in the field, and provide a structural basis to further improve the design of small sGC stimulators that could be used to treat cardiovascular diseases (Figure 1).

It remains to be resolved whether YC-1 binds to the same site in the absence of NO, since there are a number of other possible sites (Stasch et al., 2001; Lamothe et al., 2004; Wales et al., 2018). In the current study, small-angle X-ray scattering models indicate that, in the absence of YC-1, sGC enzymes with an NO-equivalent molecule bound to the heme are a mix of bent-inactive and partially extended-activated structures. However, it is still unclear how higher concentrations of NO induce the conversion to the fully extended-activated structure. The mechanism of deactivation also remains unknown. Resolving these two matters is a priority for researchers working on the physiological role of NO.
